# Dynamical Boolean Modeling of Immunogenic Cell Death

**DOI:** 10.3389/fphys.2020.590479

**Published:** 2020-11-12

**Authors:** Andrea Checcoli, Jonathan G. Pol, Aurelien Naldi, Vincent Noel, Emmanuel Barillot, Guido Kroemer, Denis Thieffry, Laurence Calzone, Gautier Stoll

**Affiliations:** ^1^Institut de Biologie de l'ENS (IBENS), Département de Biologie, École Normale Supérieure, CNRS, INSERM, Université PSL, Paris, France; ^2^Equipe 11 labellisée par la Ligue Nationale contre le Cancer, Centre de Recherche des Cordeliers, INSERM U1138, Université de Paris, Sorbonne Université, Institut Universitaire de France, Paris, France; ^3^Metabolomics and Cell Biology Platforms, Gustave Roussy Cancer Campus, Villejuif, France; ^4^Institut Curie, PSL Research University, Paris, France; ^5^INSERM, U900, Paris, France; ^6^MINES ParisTech, PSL Research University, CBIO-Centre for Computational Biology, Paris, France; ^7^Pôle de Biologie, Hôpital Europeen Georges Pompidou, AP-HP, Paris, France; ^8^Suzhou Institute for Systems Biology, Chinese Academy of Sciences, Suzhou, China; ^9^Department of Women's and Children's Health, Karolinska University Hospital, Stockholm, Sweden

**Keywords:** logical modeling, immunogenic cell death, antitumor immune response, dendritic cells, cytotoxic CD8^+^ T lymphocytes

## Abstract

As opposed to the standard tolerogenic apoptosis, immunogenic cell death (ICD) constitutes a type of cellular demise that elicits an adaptive immune response. ICD has been characterized in malignant cells following cytotoxic interventions, such as chemotherapy or radiotherapy. Briefly, ICD of cancer cells releases some stress/danger signals that attract and activate dendritic cells (DCs). The latter can then engulf and cross-present tumor antigens to T lymphocytes, thus priming a cancer-specific immunity. This series of reactions works as a positive feedback loop where the antitumor immunity further improves the therapeutic efficacy by targeting cancer cells spared by the cytotoxic agent. However, not all chemotherapeutic drugs currently approved for cancer treatment are able to stimulate bona fide ICD: some commonly used agents, such as cisplatin or 5-fluorouracil, are unable to activate all features of ICD. Therefore, a better characterization of the process could help identify some gene or protein candidates to target pharmacologically and suggest combinations of drugs that would favor/increase antitumor immune response. To this end, we have built a mathematical model of the major cell types that intervene in ICD, namely cancer cells, DCs, CD8^+^ and CD4^+^ T cells. Our model not only integrates intracellular mechanisms within each individual cell entity, but also incorporates intercellular communications between them. The resulting cell population model recapitulates key features of the dynamics of ICD after an initial treatment, in particular the time-dependent size of the different cell types. The model is based on a discrete Boolean formalism and is simulated by means of a software tool, UPMaBoSS, which performs stochastic simulations with continuous time, considering the dynamics of the system at the cell population level with appropriate timing of events, and accounting for death and division of each cell type. With this model, the time scales of some of the processes involved in ICD, which are challenging to measure experimentally, have been predicted. In addition, our model analysis led to the identification of actionable targets for boosting ICD-induced antitumor response. All computational analyses and results are compiled in interactive notebooks which cover the presentation of the network structure, model simulations, and parameter sensitivity analyses.

## 1. Introduction

**Figure 1:**
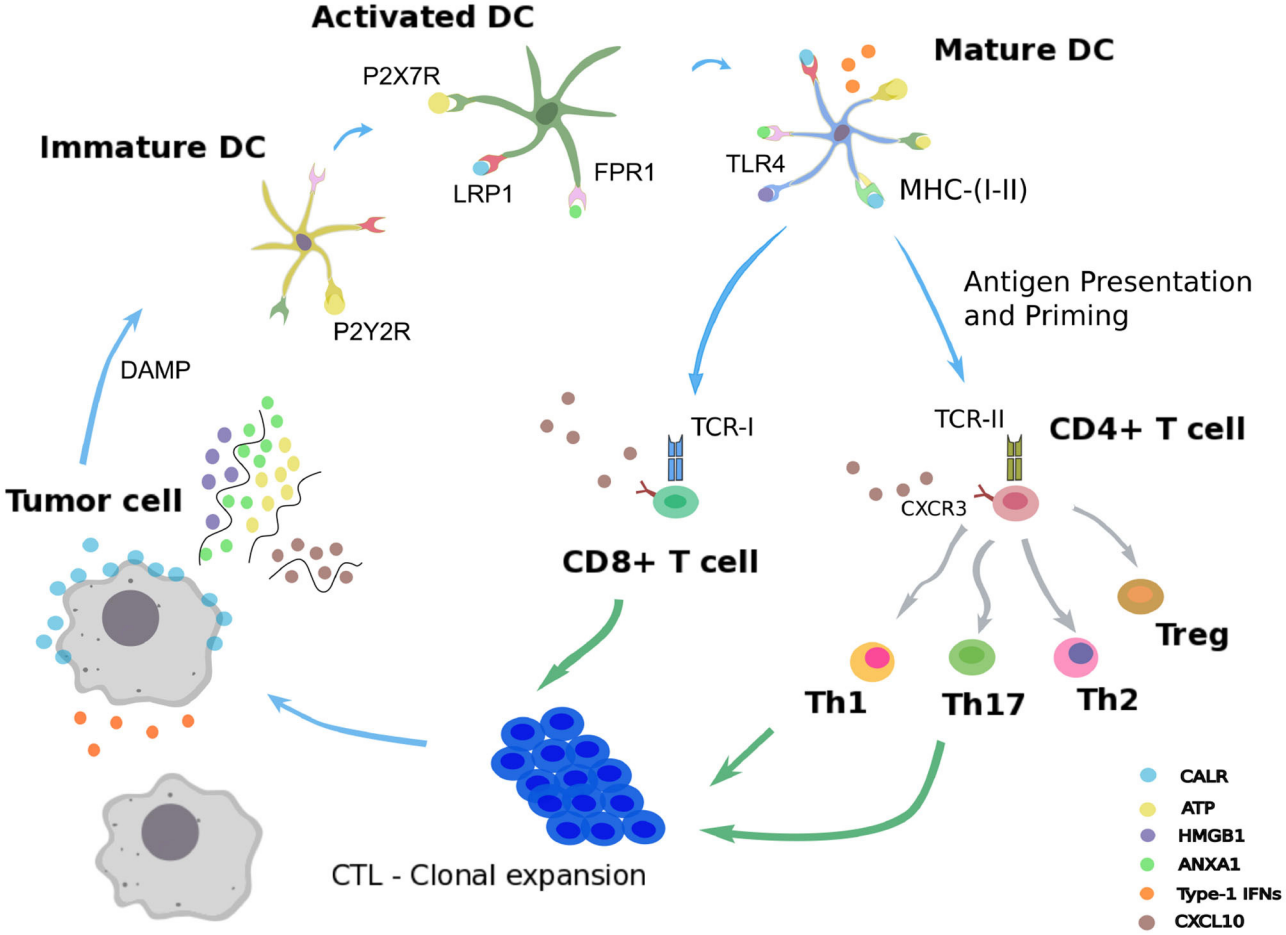
Immunogenic cell death. Schematic representation of the immunogenic cell death cycle, starting with release of DAMPs from a dying tumor cell, leading to the maturation of a dendritic cell (DC), ultimately activating CD4^+^ and CD8^+^ T cells, which in turn trigger the death of the remaining live tumor cells.

In order to ensure tissue homeostasis, programmed cell death modalities such as intrinsic apoptosis normally eliminates tumorigenic cells. When such initial safety mechanisms have failed, the immune system intervenes as a backup system to eliminate or control carcinogenic lesions (Galluzzi et al., 2017). Innate and adaptive immune actors, embodied by natural killer cells and cytotoxic CD8^+^ T cells, can mediate either extrinsic apoptosis or lysis of the aberrant cellular targets (Kroemer et al., 2013; Galluzzi et al., 2017). However, under immune pressure, some malignant cells able to evade immunosurveillance can be selected, proliferate, and generate a tumor mass. Yet, some cancer treatments, including several chemotherapies, radiotherapy, or oncolytic virotherapy, have the ability to reinstate cancer immunosurveillance (Galluzzi et al., 2017; Kepp et al., 2018). These cytotoxic interventions trigger a particular demise of transformed cells called “immunogenic cell death” (ICD) (Galluzzi et al., 2017; Kepp et al., 2018). As opposed to standard apoptosis, which is tolerogenic, ICD refers to an apoptotic process that elicits an adaptive immune response against tumor cells (Figure 1).

Following administration of an ICD-inducing treatment, dying cancer cells expose or release damaged-associated molecular patterns (DAMPs) together with tumor antigens in the tumor microenvironment (Fucikova et al., 2015; Galluzzi et al., 2017). The presence of such stress signals in the extracellular milieu allows the recruitment and activation of antigen-presenting cells, such as dendritic cells (DCs) (Galluzzi et al., 2017; Giovanelli et al., 2019). Counting among the DAMPs, extracellular ATP can be sensed by DCs through the purinergic receptors P2RX7 and P2RY2, and triggers their migration to the tumor bed and their activation (Elliott et al., 2009; Saez et al., 2017). An additional stress signal recorded upon ICD consists of calreticulin (CALR), a highly conserved chaperone protein residing in the lumen of the endoplasmic reticulum (ER) (Panaretakis et al., 2009). Upon stress of the ER, CALR translocates at the surface of the plasma membrane where it is detected by immature DCs through their low-density lipoprotein receptor-related protein 1 (LRP1) (Gardai et al., 2005). Interaction of CALR with LRP1 acts as an “eat-me” signal, which promotes tumor antigen uptake by immature DCs (Galluzzi et al., 2017; Kepp et al., 2018). The high molecular group B1 protein (HMGB1) is the most abundant non-histone chromatin-binding protein. While restricted to the nucleus in normal condition, HMGB1 is freed in the tumor microenvironment upon ICD, thereby constituting another “alarm” signal for the immune system (Dumitriu et al., 2007). HMGB1 is detected by DCs through different receptors, mainly toll-like receptor 4 (TLR4), and promotes their activation (Apetoh et al., 2007). Similarly, cancer cells undergoing ICD release the cytoplasmic protein annexin A1 (ANXA1), another DAMP that binds to formyl peptide receptor 1 (FPR1) at the surface of DCs (Vacchelli et al., 2015; Galluzzi et al., 2017; Kepp et al., 2018). Local detection of ANXA1 by DCs contributes to their homing at proximity of dying malignant cells. Finally, ICD mimics a viral infection in cancer cells by triggering the production of type 1 interferons (IFN1). These latter act in an autocrine and paracrine manner to stimulate the secretion of the chemokine C-X-C motif chemokine ligand 10 (CXCL10) by cancer cells and thus favor the recruitment of activated T cells expressing its receptor, C-X-C motif chemokine receptor 3 (CXCR3) (Galluzzi et al., 2017; Kepp et al., 2018).

Once recruited to the tumor bed by ICD-related DAMPs, activated DCs can capture tumor antigens and undergo maturation. Thus, DCs upregulate the surface class-I and class-II major histocompatibility complex molecules (MHC-I, MHC-II) and cross-present tumor epitopes onto them (Galluzzi et al., 2017; Giovanelli et al., 2019). Additionally, DCs express co-stimulatory molecules, including CD40, CD80, and CD86, and secrete inflammatory cytokines, such as interleukin-12 (IL-12), IL-6, and tumor necrosis factor alpha (TNF-α) (Galluzzi et al., 2017; Giovanelli et al., 2019). In parallel, DCs upregulate the lymphoid tissue-residing C-C chemokine receptor type 7 (CCR7) that promote their migration to the draining lymph node (Riol-Blanco et al., 2005; Galluzzi et al., 2017). In the lymph node, mature DCs prime both naive CD4^+^ and CD8^+^ T lymphocytes that display the cognate T cell receptors (TCRs) (Galluzzi et al., 2017). Activated CD4^+^ T cells can differentiate into conventional T helper cells (Th) or into regulatory T cells (Treg) (Li and Rudensky, 2016; Zhu, 2018). Depending on the cytokines locally present, several Th lineages can be distinguished, such as Th1 and Th17, which are involved in cancer immunosurveillance (Zhu, 2018). By producing type-1 cytokines such as interleukin-2 (IL-2) and IFN-γ, Th1 CD4^+^ T cells actually support the differentiation of activated CD8^+^ T cells (preCTL) into type 1 cytotoxic CD8^+^ T lymphocytes (Tc1 / CTLs), which play a critical role in eliminating malignant entities (Kurokawa et al., 2001). Activated Th1 lymphocytes and CTLs are able to migrate from the lymph node to the blood stream and eventually reach the tumor site in a CXCL10-dependent manner (Galluzzi et al., 2017; Kepp et al., 2018). In the tumor site, Th1 cells can mediate antitumor activity via the secretion of the effector cytokine IFN-γ and the CTL-mediated release of the pore-forming perforins and cytotoxic granzymes (Kepp et al., 2018; Farhood et al., 2019). This series of reactions triggered by cancer ICD form a positive feedback loop where the mounted antitumor immunity further improves the therapeutic efficacy by targeting cancer cells spared by the cytotoxic agent (Galluzzi et al., 2017; Kepp et al., 2018; Farhood et al., 2019).

In this study, our aim was to develop a model integrating the molecular and cellular entities involved in cancer ICD, together with the subsequent immune cascade resulting into antitumor activity. A better characterization of the process could help identify actionable molecular components and thus suggest combinations of pharmacological compounds that would favor/increase anticancer immunity. In particular, not all chemotherapeutic drugs currently approved for the care of cancer are able to stimulate *bona fide* ICD (Galluzzi et al., 2017). Some commonly used agents, such as cisplatin or 5-fluorouracil, fail to activate some features of ICD (Bezu et al., 2015). Nevertheless, experimental complementation of cisplatin with cardiac glycosides resulted in *bona fide* ICD and translated into potent immunotherapeutic efficacy (Kepp et al., 2012; Menger et al., 2012). An *in silico* model could accelerate the identification of such combination regimens.

To this end, we have assembled a mathematical model covering the major cell types and biological processes intervening in ICD. In order to preserve the feasibility of the simulations, we properly restricted the number of interactions and processes. Tumor cells, dendritic cells, CD4^+^ and CD8^+^ T lymphocytes have been selected as the main players. Our model focuses on intercellular communications between the different cell types, considers current knowledge on the timing of events, and takes into account death and proliferation of tumor cells in diverse contexts.

The model is based on a discrete Boolean formalism and is simulated by means of a software tool, UPMaBoSS, which performs stochastic simulations with continuous time, considering the dynamics of the system at the cell population level (an extended presentation of UPMaBoSS is provided in Stoll et al., 2020). The Boolean description of the model entities is a strong approximation. In the present work, it fits the current biological knowledge, which is mainly qualitative. However, the grammar of MaBoSS allows to represent discrete levels of each model entity, corresponding to different concentration levels or different status of a protein (e.g., phosphorylated, or in complex) entailing different levels of activity. An example of multi-level dynamics of the protein p53 in the context of p53/Mdm2 interaction is provided at the following address: https://github.com/sysbio-curie/MaBoSS-env-2.0/tree/master/tutorial/MaBoSS-2.0.

The resulting cell population model recapitulates key features of the dynamics of ICD after an initial treatment, in particular the time-dependent size of the different types of cell populations. Furthermore, the time scales of some of the processes involved in ICD, which are challenging to measure experimentally, have been predicted by proper simulations. In addition, the analysis of our model led to the identification of potential target components to modulate in order to boost ICD-induced antitumor response.

## 2. Materials and Methods

For modeling ICD, we use the stochastic Boolean simulation framework UPMaBoSS (Stoll et al., 2020). It is based on the previously defined MaBoSS grammar (Stoll et al., 2012), which was extended to simulate the dynamics of the cell populations. Details about the software are provided below.

More information about software accessibility, use cases, and jupyter notebooks including the code used for all the analyses presented here are available on a GitHub repository: https://github.com/sysbio-curie/ICD.

### 2.1. Cell Population Described as a Probabilistic Boolean System

**Figure 2:**
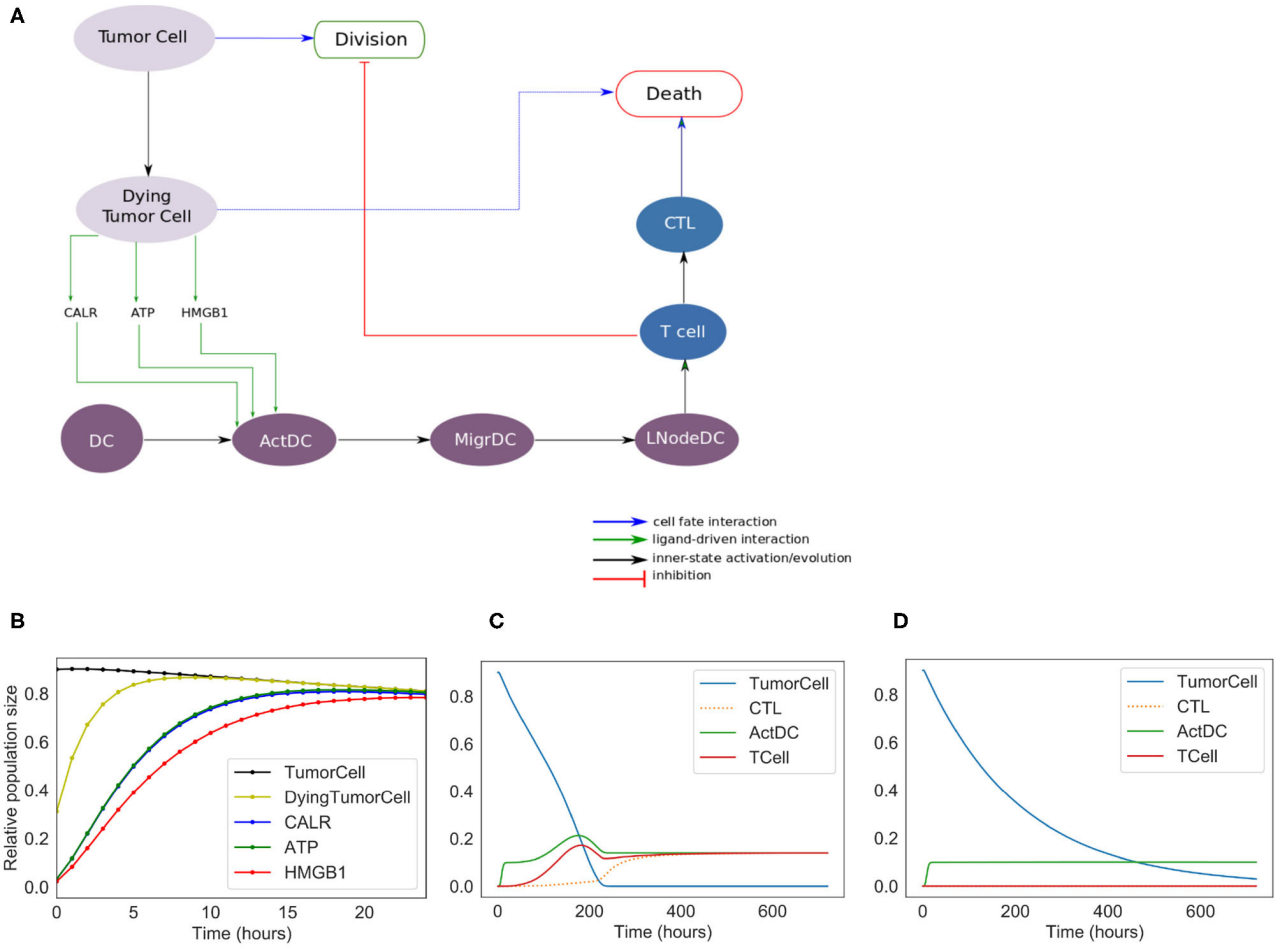
Phenomenological model. **(A)** Influence network of the simplified version of ICD: it involves three cell types: tumor cells (Tumor Cell and Dying Tumor Cell for cells that have been treated by chemotherapeutic agents, gray nodes), dendritic cells (DC for immature dendritic cells, ActDC for mature dendritic cells, MigrDC for migrating dendritic cells, and LNodeDC for dendritic cells in the lymph node, purple nodes), T cells (T Cell, and CTL for cytotoxic T lymphocyte, blue nodes). **(B)** Early activation of ICD markers (Note: CALR overlaps with ATP). **(C)** Kinetics of the cell types with ICD. **(D)** Kinetics of the tumor cell populations without ICD.

The model is based on a regulatory network (directed graph), where nodes represent molecules, cell types and processes, whereas arcs represent positive or negative influences between these entities. In this application, some arrows can also represent transformation of cells. For instance, an immature DC can become mature (example in Figure 2, from DC to ActDC). In the Boolean description, the variables corresponding to these entities can take two values, 1 for active or present, and 0 for inactive or absent. We define a *network state* as a set (or a vector) of Boolean values associated with each of these entities. We apply a probabilistic framework to these network states. More precisely, a probability is associated with each network state, and the sum of probabilities over all possible network states is equal to 1. The interpretation in terms of population is straightforward: the number of cells in a given network state is equivalent to the network state probability multiplied by the overall size of the cell population.

### 2.2. Signaling Pathways Described as MaBoSS Models

MaBoSS (Stoll et al., 2012, 2017) is a software dedicated to the modeling of signaling pathways based on a Boolean probabilistic description. Given a Boolean model, MaBoSS produces time dependent probabilities of network states. In MaBoSS, two transition rates (activation/inhibition) are associated with each node of the network. These rates are written in a specific grammar, which combines Boolean states of other nodes with logical (AND, OR, NOT) and arithmetic operators. In addition to this set of transition rates, a MaBoSS simulation requires the definition of an initial condition, i.e., a probability distribution of network states at time zero. In practice, a MaBoSS model is encoded into two files: a *bnd* file containing the logical rules and the definition of the transition rates, and a *cfg* file containing the parameters of the model (initial conditions and values for the transition rates) together with the simulation parameters.

### 2.3. Population Dynamics Described as an UPMaBoSS Model

MaBoSS can only model a cell population composed of non-interactive cells, with a constant population size. In order to better represent the dynamics of the cell populations, we recently developed an extension of MaBoSS, called UPMaBoSS (Stoll et al., 2020). In this framework, cells can divide, die and interact. In this respect, two additional nodes are added to the usual MaBoSS model: *Death* and *Division*. Regarding cell communication, ligand-receptor interactions are introduced: the transition rates of the receptors can contain parameters that are updated according to the state of the whole cell population. At regular time steps, the simulation is stopped and some variables are updated according to their status. Cells having the *Division* node at 1 are doubled, whereas those having the *Death* node at 1 are removed. Receptors are updated according to the value of their regulators.

An UPMaBoSS model is encoded into three files: *bnd* and *cfg* files written in MaBoSS grammar, completed by a specific *upp* file. The *upp* file contains the declaration of the *Death* and *Division* nodes, together with the updating rules for the parameters controlling the transition rates of the receptors.

All simulations of the ICD models reported here are encoded in the jupyter notebooks provided as Supplementary Material. These notebooks can be executed on any computer with a recent docker or a conda environment installed, as indicated in the dedicated GitHub repository (https://github.com/sysbio-curie/ICD). The full parameter sensitivity analysis is not included in the jupyter notebooks, as it is time consuming (taking over 24 h) and has been performed on a computer cluster. Results of the computations are available in Supplementary Tables 4, 5. The notebooks contain some examples of models with modified parameters.

### 2.4. Phenomenological Model

The purpose of the ICD phenomenological model is to reproduce the succession of events observed experimentally, and to determine the role of each of the main cell types involved in ICD. This model serves as a basis to develop a more detailed, “extended” model. The phenomenological model includes three cell types with different status (Figure 1). *Tumor Cell* and *DC* constitute the inputs of the model. The simulation starts with a predefined tumor size and a given population of DCs. Tumor cells can die or divide. Dying tumor cells release some danger signals (CALR, ATP, and HMGB1), which activate DC (*ActDC*), which can then migrate (*MigrDC*) to the tumor draining Lymph Node (*LNodeDC*). There, mature DCs can activate T cells. T cells can then differentiate into cytotoxic effectors (*CTL*), proliferate, and reach the tumor through blood vessels. In contact with a CTL, tumor cells are cleared out. Without treatment or in the absence of T cells, tumor cells keep proliferating. The corresponding model files can be found at: https://github.com/sysbio-curie/ICD. The description of the model with the meaning of the variables and the parameter values are detailed in the Supplementary Table 1.

### 2.5. Extended Model

Some modeling choices were made to refine the phenomenological model, while preserving the overall dynamics. This extended model includes detailed representations of the series of events previously explored with the phenomenological model. The abstract transitions considered in the initial model are replaced by more refined details about which and how cells interact with ligand-receptor dynamics. Some of the ligands depend on the status of the cell type that produces them, and/or on the activity (or availability) of a receptor that mediates their activation. The transition rates associated with ligand activation are usually set as the inverse of the time that a ligand takes to reach its concentration peak.

**Figure 3:**
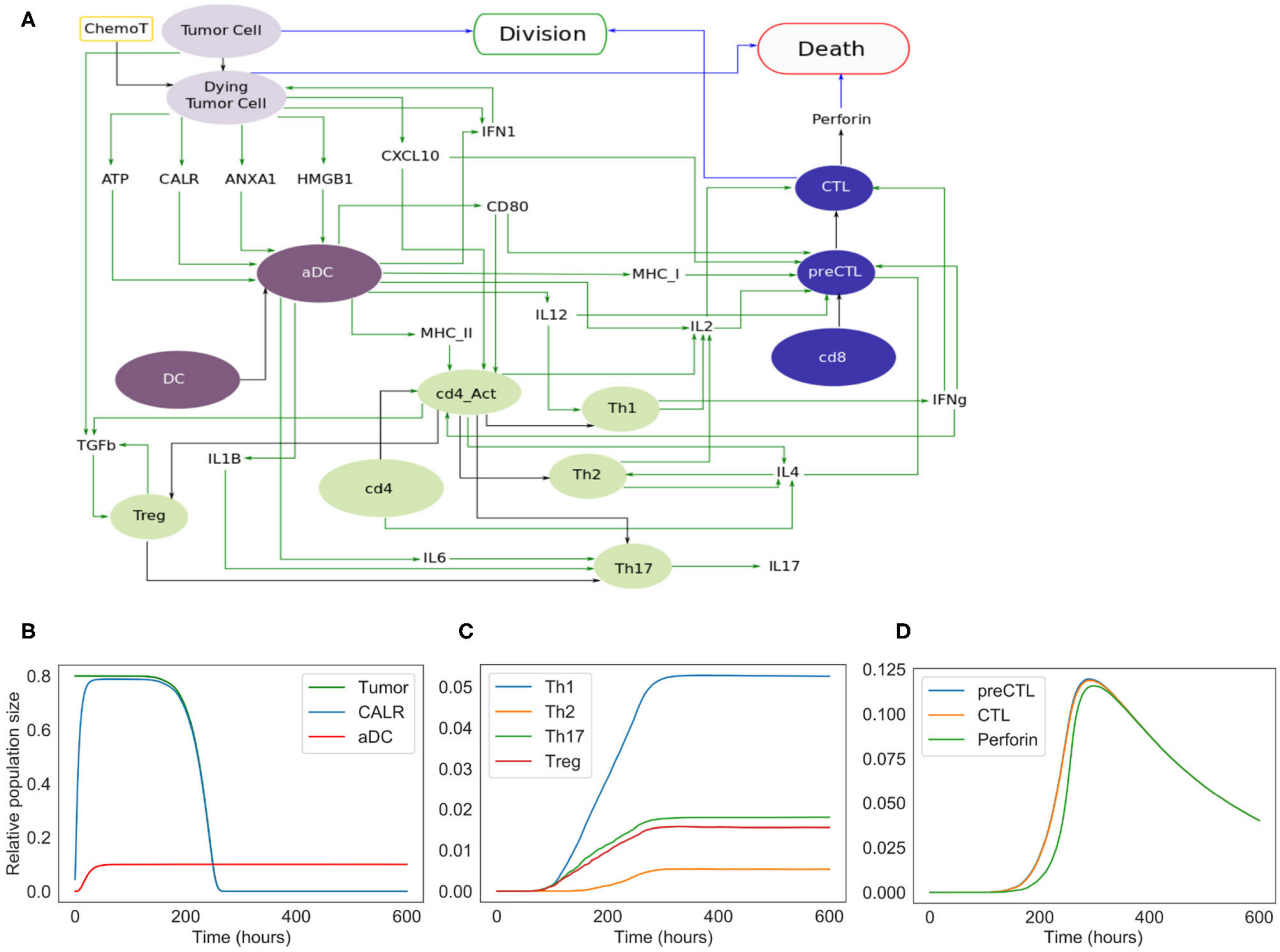
**Extended model. (A)** Influence network of the extended version of ICD involving four cell types: tumor cells (light purple nodes), dendritic cells (dark purple nodes), CD4^+^ T cells (green nodes) and CD8^+^ T cells (blue nodes). Green arcs correspond to activation, black arcs to cell transformation, and blue arcs to cell fate interactions. Tumor Cell, DC, cd4, and cd8 are inputs. When the fifth input, namely ChemoT, is active, we consider that cells have incorporated a chemotherapeutic drug. The two cell fates, Division and Death, are outputs of the model. **(B)** Kinetics of the populations of tumor and active dendritic cells, as well as of CALR surface exposure. **(C)** Kinetics of the helper and regulatory CD4^+^ T cell subpopulations. **(D)** Kinetics of the CD8^+^ T cell subpopulations.

In this extended model, we consider four cell types, including tumor cells, dendritic cells, CD4^+^ T cells, and CD8^+^ T cells, as shown in Figure 3. As for the phenomenological model, the size of the populations of these four cell types must be defined at the beginning of a simulation.

The extended model encompasses 57 entities, with some entities that may correspond to different instances of the same species or cell types (e.g., DC and aDC where “a” stands for active). Figure 3 includes the cell types and their fate: *Tumor Cells* can die under treatment and become *Dying Tumor Cells*, dendritic cells (*DC*) can be activated in the presence of ATP, CD4^+^ cells (*cd4*) can lead to Th1, Th2, Th17, or Treg cells, and finally CD8^+^ cells (*cd8*) become *CTL*.

The cytokines and the corresponding receptors involved in ICD are each represented by a specific node (denoted in the model by *-o for a cytokine, and by *-rec for a receptor). The activation rule for each cytokine relies on the cell type producing it and possibly on the adjuvant effects of other ligands, enhancing the activation of such cytokines. On the other hand, a cytokine receptor node is conditioned by the presence of the cells presenting it. The two types of nodes, ligand and receptor, are dynamically linked, as the probability attributed to the ligand node impacts the rate of transition toward the activation of the receptor node. At each step of the simulation, after its first activation, the transition rate for the receptor is controlled by the ligands.

All the logical rules are written to account for the biological knowledge whenever it is reported in the literature. For instance, the rule for the CD8^+^ T cell receptor node *TCR-I* is: CD28 & CD8, which reads as: when both CD28 (the receptor of CD80) and a naive CD8^+^ T cell are present, TCR_I will be activated at a rate $u_TCR_I$, which is defined in the *cfg* file.

The model files can be found at: https://github.com/sysbio-curie/ICD, the model description with the meaning of the variables and the parameter values are detailed in Supplementary Table 2, and the list of logical rules are provided in Supplementary Table 3.

## 3. Results

To simulate the different steps of ICD, we considered two models: the *Phenomenological* and the *Extended* models. The first model contains ICD markers and a minimal number of cell types. It is easy to handle because of its limited size and serves as a basis for the more detailed version, the *Extended* model.

### 3.1. Phenomenological Model of ICD

We constructed a simplified model of ICD, focusing on a few key cell types and their interactions (Figure 2). This model contains 13 nodes (therefore 2^13^ = 8192 possible states) and 19 transition rate parameters (see Supplementary Material). The nodes of the regulatory network are each associated with a Boolean variable, and transition rates account for the timing of events.

In the regulatory graph shown in Figure 2A, the ellipses represent the different cell types considered: Tumor cell, Dying Tumor Cell, Dendritic Cell (DC), Activated Dendritic Cell (ActDC), Migrating Dendritic Cell (MigDC), Lymph Node Dendritic Cell (LNodeDC), T Cell (TCell), and Cytotoxic T Lymphocyte (CTL).

To keep the model as simple as possible, the network contains numerous shortcuts and over-simplifications: for instance, the arc from LNodeDC to TCell in Figure 2A represents the fact that a single dendritic cell usually activates a single T Cell.

We introduce different successive status for the dendritic cells (Activated, Migrating, in the Lymph Node), which delay T cell activation. This insures that T cells do not activate immediately after the activation of DC by the ICD-emitted danger signals (CALR, ATP, and HMGB1).

Out of the 19 parameters of this model, 14 must be set manually (the 5 others are updated at the population level and their initial values are set to 0). As the timing of ICD can vary between experimental models, we decided to choose values that have the correct order of magnitude and reproduce the expected timing of ICD (these parameter values are given in Supplementary Table 1). More specifically, we set the mean time of “activation” of CALR and ATP at 4 h, and of HMGB1 at 6 h post-ICD inducing chemotherapy (Fucikova et al., 2011). Additionally, we set at “low” the direct cytotoxic efficacy of the treatment, the mean time of DC migration from the tumor to the lymph nodes at 5 days, the mean time of division of tumor cells and CTLs at 10 and 1/2 day(s), respectively, and the mean time of differentiation of T Cells into CTLs at 2 days.

The results of the simulations of the model are shown in Figures 2B–D. Following ICD-inducing intervention, the release of CALR, HMGB1, and ATP by dying cancer cells is observable within hours (Figure 2B). After 100 h (Figure 2C), the immune system is activated with a slow increase of T cells, which peaks at 200 h. The tumor cells are cleared out at about 220 h, which coincides with an increase of the CTL population. To investigate the role of the immune system in the disappearance of the tumor cells, we removed the clonal expansion of the CTLs in the phenomenological model, i.e., the recruitment of immune cells following chemotherapy. In the absence of such increase of the immune effector population, tumor cell clearance becomes less efficient since it relies mostly on the direct cytotoxicity of the treatment (Figure 2D).

The phenomenological model successfully reproduces the series of events that are associated with ICD and leading to tumor cell killing following immunogenic chemotherapy. However, the predictive power of this model remains limited because of the lack of molecular details. Hence, we decided to extend this model by further detailing the molecular intermediates, with a focus on the intercellular dialogues.

### 3.2. Extended Model of ICD

To improve our phenomenological model, we introduced additional nodes representing molecular factors. More precisely, variables such as *MigrDC* and *LNodeDC*, accounting for the activated dendritic cells transiting in the circulation or reaching the tumor draining lymph node, respectively, were replaced by molecules mediating these phenomena.

In this extended version of the model (Figure 3A), four populations of cells are considered: tumor cells, dendritic cells, CD4^+^ and CD8^+^ T cells. As for the phenomenological model, tumor cells can be converted into dying tumor cells when treated by chemotherapeutic agents, whereas dendritic cells become active after sensing ATP, HMGB1, CALR, and/or ANXA1.

Without treatment, tumor cells proliferate indefinitely. In our model, we implicitly assume that the cells have been treated by an ICD-inducing therapy (node *ChemoT*). Also, we set the initial size of the tumor cell population. These tumor cells produce stress-induced ligands (DAMPs) (Fucikova et al., 2015): CALR, ATP, ANXA1, and HMGB1, whose activation is conditioned by several constraints. For instance, HMGB1 is ready to be released only if ANXA1 is present in the extracellular milieu.

Initially inactive and distant from the tumor bed, DCs become active (denoted by *aDC*) after the stimulation of purinergic receptors upon tumor-derived extracellular ATP binding (mainly the high-affinity metabotropic P2Y2R and the low-affinity ionotropic P2X7R; Rossi et al., 2012), which ignites the migratory status of the DC. This biological information has been translated into a Boolean rule as follows: if a DC and P2X7R are both active at the same time, then DC switches to its active state. Interaction between the activated DC and CALR at the surface of the dying tumor cell triggers its phagocytosis, thereby promoting tumor antigen uptake by the DC (Galluzzi et al., 2017).

Along their way to the lymph node, activated DCs (*aDCs*) capturing antigens upregulate MHC (class-I and II) molecules together with co-stimulatory molecules such as CD80. Once in the secondary, or eventually tertiary, lymphoid tissue, the encounter between such mature DC presenting tumor antigens and a naive undifferenciated T lymphocyte can lead to an activated T cell (Zehn et al., 2012). T cell activation occurs when the cognate TCR and CD28, that are exposed on the lymphocyte, interact with the antigen-loaded MHC and CD80 at the surface of mature DCs, respectively (Galluzzi et al., 2017; Patente et al., 2018). The transition rate associated with the activation of each receptor node is function of the state of the ligand to which it binds. Thus, the activity of the CD8^+^ T cell receptor (labeled “TCR-I”) node is affected by the activity of the MHC-I node. The more MHC-I is produced (i.e., high activation probability), the more likely it is to activate TCR-I.

CXCR3 is a chemotactic receptor on activated T cells, which binds CXCL10 released by tumor cells and by intratumoral activated DCs, following autocrine and paracrine stimulation by type-1 IFNs (Figure 3) (Zitvogel et al., 2015; Galluzzi et al., 2017). When CD28 is active, we are considering a single T cell (either CD4 or CD8) whose CXCR3 receptors have already been activated. Further details regarding these interactions are provided in the Supplementary Figures 1, 2.

Activated DCs not only secrete type-1 IFNs but also additional ligands such as the cytokines IL-6 and IL-12, which impact the differentiation of naive T cells as detailed below (Figure 3) (Henry et al., 2008; Subbiah et al., 2018).

For the sake of simplicity, we considered a CD4^+^ T cell as activated (denoted by *cd4_Act* ON) when a lymphocyte expressing both CD28 and a “TCR-II” (i.e., a TCR that recognizes a cognate MHC-II associated with an antigen epitope) interacts with a mature DC (Zehn et al., 2012; Chen and Flies, 2013). Following activation, CD4^+^ T cells differentiate into effector or regulatory subtypes, depending on the cytokines locally present. Of note, the memory compartment, including the central and effector memory T cell subsets, is not taken into consideration in our current model. Therefore, in the presence of DC-produced IL-12, CD4_Act will differentiate into Th1 CD4^+^ T cells. By contrast, the sensing of T cell-produced IL-4 induces the Th2 program. Alternatively, the detection of IL-6, TGFb as well as IL1B promotes Th17 differentiation. Also, binding of TGFb engages the undifferenciated lymphocytes into the immunosuppressive Treg lineage (Figure 3A).

To account for the fact that Th0 cells do not solely lead to Th1 or Th2 cells, we included Th2, Th17 and Treg subtypes, even though their respective role in the series of immune reactions that follow cancer ICD remains poorly characterized (Galluzzi et al., 2017).

It has been reported that IL-4 is produced by either Th0, Th2 or precursors of CD8^+^ cytotoxic T lymphocytes (CTLs) (see below for details about CD8^+^ T cell subtypes) (Zhu, 2015; Farhood et al., 2019). The activation (or release) of this interleukin is thus conditioned by the presence of either cell type.

This means that a naive CD4^+^ can be driven into proliferation by sensing IL-4, while Th2 is able to sustain its activity.

In parallel, naive CD8^+^ T lymphocytes can be primed (i.e., co-stimulation of TCR-I and CD28) by mature DCs presenting tumor antigen epitopes onto MHC-I molecules and turned into cytotoxic precursors (*preCTL*). Through the release of both IL-2 and IFNg, Th1 lymphocytes further support the differentiation of preCTL into CTLs (Galluzzi et al., 2017; Farhood et al., 2019). preCTL can be activated by the co-stimulation of MHC-I (represented by the receptor TCR-I in the model) and in the presence of IL-2. They turn into CTL under the influence of IFNg or that of IL-2. While IL-2 is produced by several cell types including mature DCs, Th0, Th1, and Th2, IFNg is only produced by Th0, Th1 and CTLs under the combined effect of several cytokines that enhance its production (Bhat et al., 2017).

CTLs can then release perforin, a cytolytic protein able to form pores in target cells and allow pro-apoptotic proteases to initiate cell death (Halle et al., 2016). Intracellular granules of perforins can be replenished allowing CTLs to kill more than one target tumor cell. If tumor cells can die following a chemotherapeutic treatment, they can also be efficiently eliminated by CTLs excreting perforins. It is modeled here by an amplification factor which ensures a rapid decrease of the remaining tumor cells.

The extended model contains 98 parameters. Among them, 20 are updated at the population level and are initially set to 0. The parameter values are listed into the Supplementary Table 2. As for the phenomenological model, not all the parameter values are found in the literature; thus, most of them were set to reasonable values with a correct order of magnitude. Specifically, the mean activation time of proteins have been set to 6 h, except for ATP and CALR, for which this parameter is set to 4 h, according to the literature (Liu et al., 2019; Turubanova et al., 2019; Galluzzi et al., 2020; Humeau et al., 2020), and the mean degradation time of proteins is set to 12 min. We also considered a short activation time of 12 min for the cell types: aDC, Th1, Th2, Th17, Treg, preCTL, as the timing is controlled by proteins. This is not the case for CTL activation, whose mean activation time is in the order of 2.4 h. We consider a very slow growing population of tumor cells (mean time of division is 100, 000 h), and the half-life of the chemotherapeutic agent was estimated at 3 days in the tumor tissue (An and Morris, 2012).

ICD induction was considered to occur 1 h after delivery of the pharmaceutical compound, as it needs 50, 000 h for a tumor to be cleared out by direct cytotoxicity of chemotherapy. We chose a slow growing population of tumor cells and a slow direct cytotoxicity of chemotherapy in order to focus the model on ICD.

During clonal expansion, a lymphocyte needs 20 h to divide. If a tumor cell is completely surrounded by CTLs with perforins, it dies within 1 h. If a CTL with active perforin is completely surrounded by tumor cells, it will loose its perforins in 6 h (but perforin may be reactivated), and will be definitively inactive after 10 days when CTL is estimated to experiment death. These latter parameters are difficult to estimate from experimental data, which justified the sensitivity analysis below.

The simulations of the extended model recapitulated the succession of events leading to ICD. The initial population is composed of 80% of tumor cells, 10% of dendritic cells, and 5% of inactive CD4^+^ and CD8^+^ cells. As shown in Figure 3D, following the chemotherapeutic treatment, cells start to die, expose CALR on their surface, and release other DAMPs (ATP, ANXA1, HMGB1), ultimately triggering DC maturation. CD4^+^ and CD8^+^ cells are then activated and are able to differentiate into T helpers and cytotoxic subtypes. The population of tumor cells undergoes a fast decay starting from 250 h (Figure 3B), corresponding to the activation of the adaptive immune response, when Th1 (as well as other subtypes), and most importantly CTLs, are engaged (Figures 3C,D). Ultimately, tumor cells are targeted and cleared out by perforins upon CTL degranulation (Figure 3D).

### 3.3. Sensitivity Analysis

We performed a sensitivity analysis to test the global robustness of the extended model. The model contains 98 parameters, but 20 of them have 0 as initial value and are updated at the population level. Therefore, the sensitivity analysis is performed on the 78 remaining parameters. In this respect, we systematically increased and decreased each parameter by 50% separately, which corresponds to 156 model variants (see Supplementary Table 4). To assess the effect of the immune system on the killing of the population of tumor cells, we plotted the tumor size at time = 220 h and time = 280 h, which corresponds to the time frame when the tumor cell population undergoes a swift decrease of its size in the initial “standard” conditions (WT) of the extended model (Figure 3B).

**Figure 4:**
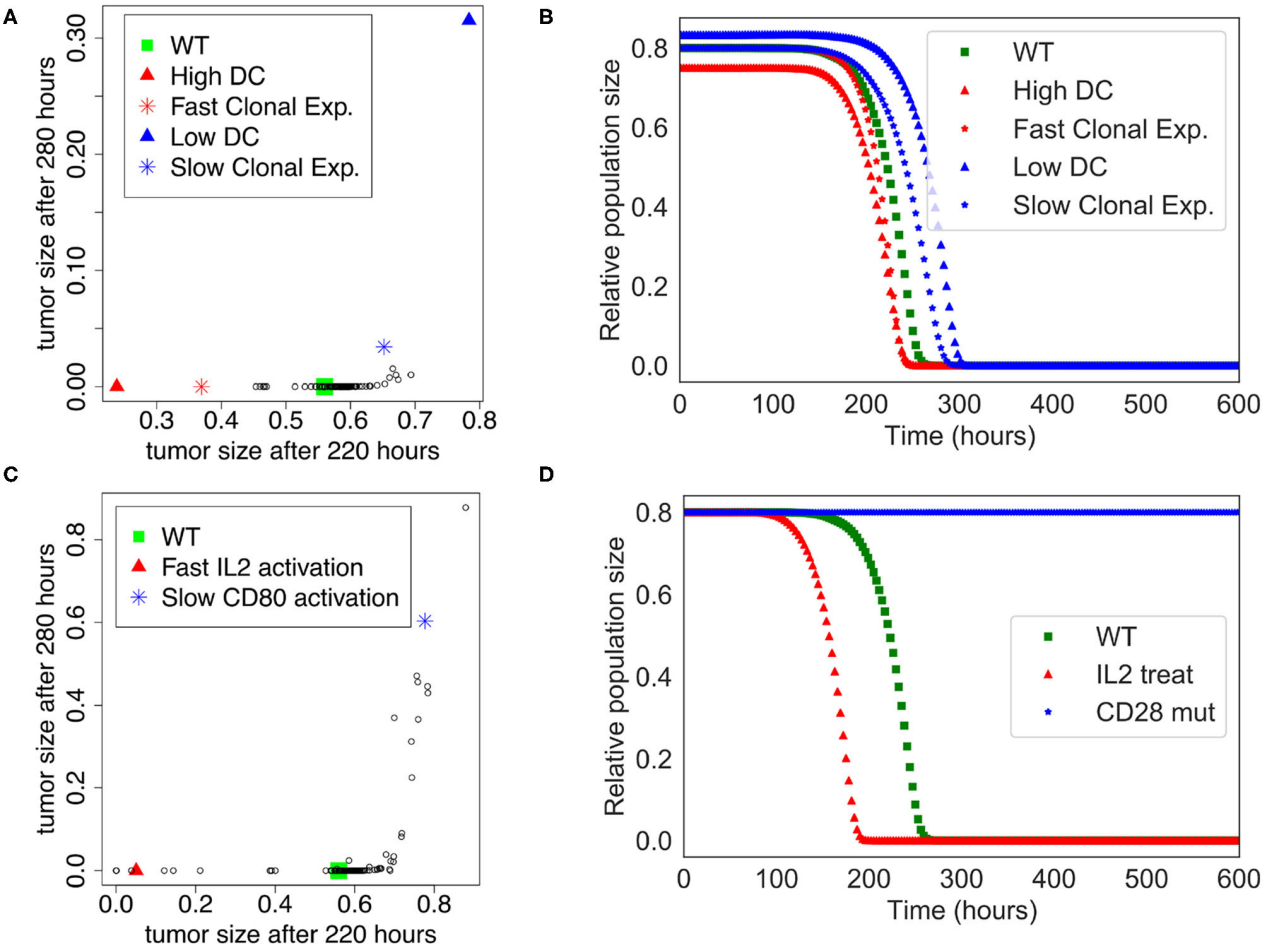
Sensitivity analysis of the Extended Model. **(A)** Tumor size at time= 220 h vs. time= 280 h when the different parameters of the extended model were increased or decreased by 50%. Initial amount of Dendritic cells and rate of clonal expansion show the strongest effect (for WT, *$InitDC*= 0.1, *$clonal*_*exp*_*rate*= 0.05; for High DC, *$InitDC*= 0.15 and Low DC, *$InitDC*= 0.067; for Fast Clonal Exp., *$clonal*_*exp*_*rate*= 0.075, and for Slow Clonal Exp., *$clonal*_*exp*_*rate*= 0.033). **(B)** Kinetics of the size of the tumor cell population according to the parameters changes highlighted in **(A)**. **(C)** Tumor size at time= 220 h vs. time= 280 h when the different parameters of the extended model were multiplied or divided by 5. Faster activation of IL2 and slower activation of CD80 have the strongest effect. **(D)** Kinetics of the size of the tumor cell population following a treatment with IL2 (IL2 treat) or a mutation of CD28 on T cells (CD28 mut), inspired by the parameters changes highlighted in **(C)**.

The sensitivity analysis shows that the model is quite robust to parameter changes. Indeed, the drop in size of the tumor cell population is slightly anticipated or delayed for a few parameter changes when compared to the WT condition (Figure 4A). For instance, the parameters that control the number of DCs are showing the strongest effect ($InitDC in WT model, and More_InitDC and Less_InitDC in the model variants): a lower amount of DCs is delaying time of death whereas a higher amount is accelerating the process. A similar effect is observed for the parameter controlling the rate of T cell clonal expansion ($clonal_exp_rate in the WT model, and More_clonal_exp_rate and Less_clonal_exp_rate in the model variants). Nevertheless, these strongest effects only affect slightly the drop of tumor size (Figure 4B). A full list of the effects observed following parameter changes can be found in the Supplementary Table 4, where the acronym “More” corresponds to +50%, and “Less” correspond to −50%.

In order to suggest possible points of intervention to further stimulate ICD, we performed the same sensitivity analysis, but by multiplying and dividing by a factor of 5 each parameter separately (Figure 4C), thus mimicking a mutation of the corresponding node. Among the strongest effects, we selected two conditions: the slower activation rate of CD80 and the faster activation rate of IL-2, labeled LLess_rate_CD80_i and MMore_rate_IL2_i, respectively (cf. Supplementary Table 5). These observations led us to simulate a complete knock out of CD28 (CD28 is the target of CD80) and an external treatment by IL-2. The results of these two modifications are shown in Figure 4D. In the case of a CD28 knock-out, we observed that 80% of the tumor cell population persists at t=280 h, sign of a failure of the ICD-inducing treatment (knowing that the tumor cell population is initially set to represent 80% of the total number of cells at the beginning of the simulation). Similarly, a treatment that would lead to an increase of IL-2 could kill the tumor cells faster at *t* = 200 h.

This analysis shows that our extended model enables the exploration of perturbations that potentiate the killing of tumor cells by boosting the adaptive immune response which follows an ICD-inducing chemotherapeutic treatment. It is important to note, though, that the approach is not quantitative, and cannot provide regimens of drug treatments, but it can highlight potential mechanisms and molecular targets that could increase tumor clearance.

## 4. Discussion

ICD of tumor cells is induced by a combination of factors and requires a cooperation between several players of the tumor microenvironment, in particular T cells and DCs. If we still lack a full understanding of the molecular mechanisms governing ICD, as well of the cross-talk between the ICD-induced immune players and the tumor cells, we believe that mathematical modeling could contribute to a more comprehensive understanding of these processes. For this purpose, we integrated information about ICD dispersed in multiple scientific papers into regulatory networks, which explicitly consider how the main cell types communicate.

More specifically, we have constructed two models with two different purposes. The first model aimed at verifying that the series of events that lead to ICD could be reproduced, and at suggesting some parameter values that could mimic the current knowledge related to the timing of these events. This phenomenological model was then used as a basis for a more complex model that included additional key molecules involved in the cascade of events associated with ICD. With the extended model, we were able to further explore the dynamics of the cell type populations subjected to different conditions (number of DCs, speed of some processes, etc.).

To do so, we used a stochastic simulation environment accounting for cell death, cell division and inter-cellular communication to monitor population sizes for different cellular conditions. Interestingly, although certainly still over-simplified, we could recapitulate several essential features of ICD with our model, and even pinpoint the roles of specific components, which might be properly acted upon to boost the immune response.

Such results could be interpreted as possible pharmacological interventions that could improve chemotherapy outcome. The model is an important and necessary tool in such a context because many of these parameters that we can explore with the model are difficult to measure experimentally. To that end, sensitivity analyses have confirmed that the model is suited for strong ICD inducers, like oxaliplatin. To contextualize the model for weak ICD inducers (like mitoxantrone), other mechanisms should be added to the model. The fact that we were only able to switch the timing of complete removal of tumors rather than to reduce the effect of the tumor removal suggests that some essential molecules are still missing in the model.

Future work will include specific *in vitro* and *in vivo* experiments in order to fit parameters to the data and match experimentally-observed timing of the different events leading to tumor clearance. We also plan to extend the model by refining some already described intercellular interactions, e.g., details about the production and effects of IFNg or TGFb on the immune cells, and also by including major signaling pathways inside each cell type to allow more candidates in the search for improving ICD.

A long-term goal is to propose feasible pharmacological interventions that can boost ICD for killing tumor cells, probably by targeting multiple elements throughout the ICD process. The first step of this approach is done with the sensitivity analysis on the refined mathematical model to identify the candidates to target. The second step requires a confirmation of the results with public omics data, a list of potential pharmacological targets, as well as *in vivo* validation, together with further pharmacodynamics/pharmacokinetics studies.

## Data Availability Statement

All datasets generated for this study are included in the article/Supplementary Material.

## Author Contributions

JP, GS, and LC conceived the project. GS, LC, and DT supervised the study. AC constructed the logical models. GK and JP provided expertise in the biological interpretation. AN, EB, and VN provided expertise in the computational biology. GS, LC, AC, and JP wrote the manuscript. AN, VN, EB, GK, and DT edited the manuscript. All co-authors agreed on the manuscript.

## Conflict of Interest

The authors declare that the research was conducted in the absence of any commercial or financial relationships that could be construed as a potential conflict of interest.

## Supplementary Material

GitHub repository at https://github.com/sysbio-curie/ICD/: 1. Jupyter notebook for phenomenological model (ICD_phenomenological.ipynb); 2. Jupyter notebook for the extended model (ICD_extend.ipynb); and 3. model files with .bnd .cfg .upp files.

Supplementary Table 1 Table of variables and parameters for the phenomenological model: Table_S1_ICD_phenomenological_description.xlsx.

Supplementary Table 2 Table of variables and parameters for the extended model: Table_S2_ICD_extended_description.xlsx.

Supplementary Table 3 Table of logical rules: Table_S3_logical_rules.txt.

Supplementary Table 4 Sensitivity analysis with all parameters increased and decreased by 50%: Table_S4_SensitTum220vs280_0.5.xlsx.

Supplementary Table 5 Sensitivity analysis with all parameters increased and decreased by 5: Table_S5_SensitTum220vs280_5.xlsx.

Supplementary Figure 1 Ligand-receptor dynamics related to dendritic cells. DC shows receptors (blue nodes) on its surface (dashed arcs) that can interact with ligands (orange nodes) and can then release ligands (pink nodes).

Supplementary Figure 2 CD4^+^ cell dynamics with description of cell differentiated lineages and the role of the cytokines in the activation of the CD4^+^ T cell dynamics. Black arcs represent activating influences and red arcs inhibiting influences.
