# Supplementary figures and images for: Dynamical Boolean Modeling of Immunogenic Cell Death
Source: Front Physiol. 2020 Nov 12;11:590479. doi: 10.3389/fphys.2020.590479 (PMC7690454; doi:10.3389/fphys.2020.590479)

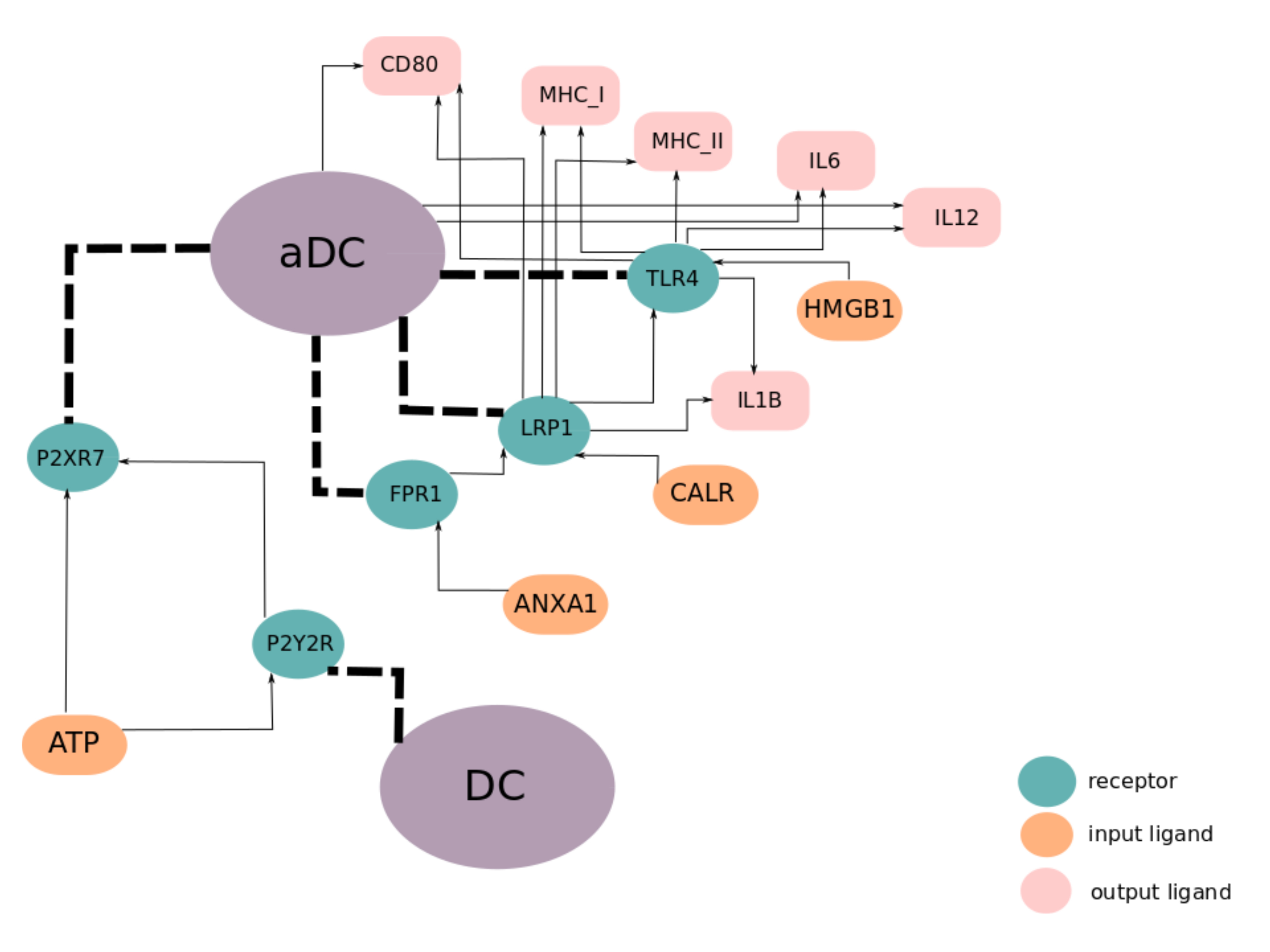

Supplement: Supplementary Figure 1 — Ligand-receptor dynamics related to dendritic cells. DC shows receptors (blue nodes) on its surface (dashed arcs) that can interact with ligands (orange nodes) and can then release ligands (pink nodes). [file Image_1.JPEG]

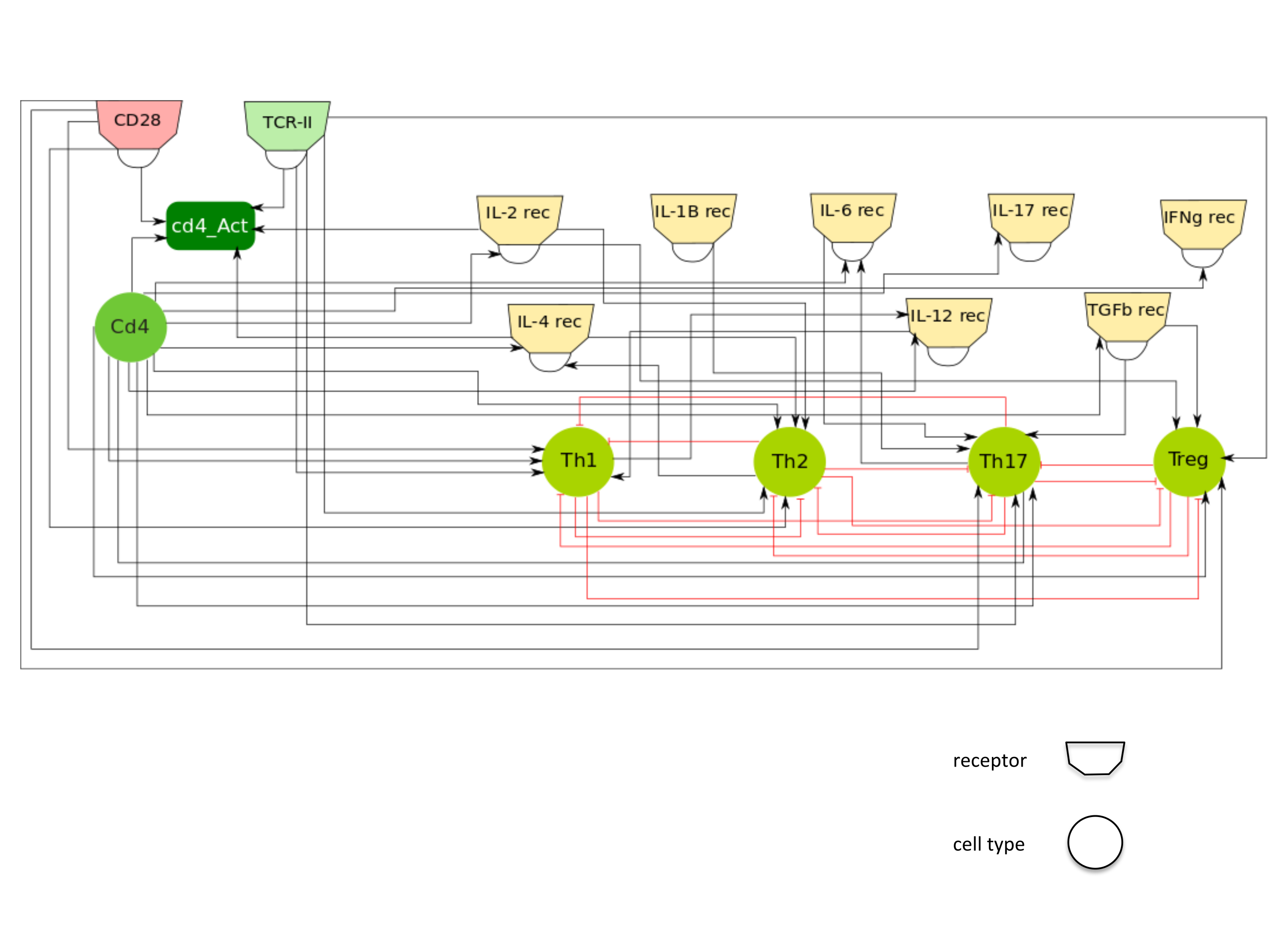

Supplement: Supplementary Figure 2 — CD4+ cell dynamics with description of cell differentiated lineages and the role of the cytokines in the activation of the CD4+ T cell dynamics. Black arcs represent activating influences and red arcs inhibiting influences. [file Image_2.JPEG]
